# Supplementary figures and images for: The Autophagic Activator GHF-201 Can Alleviate Pathology in a Mouse Model and in Patient Fibroblasts of Type III Glycogenosis
Source: Biomolecules. 2024 Jul 24;14(8):893. doi: 10.3390/biom14080893 (PMC11352067; doi:10.3390/biom14080893)

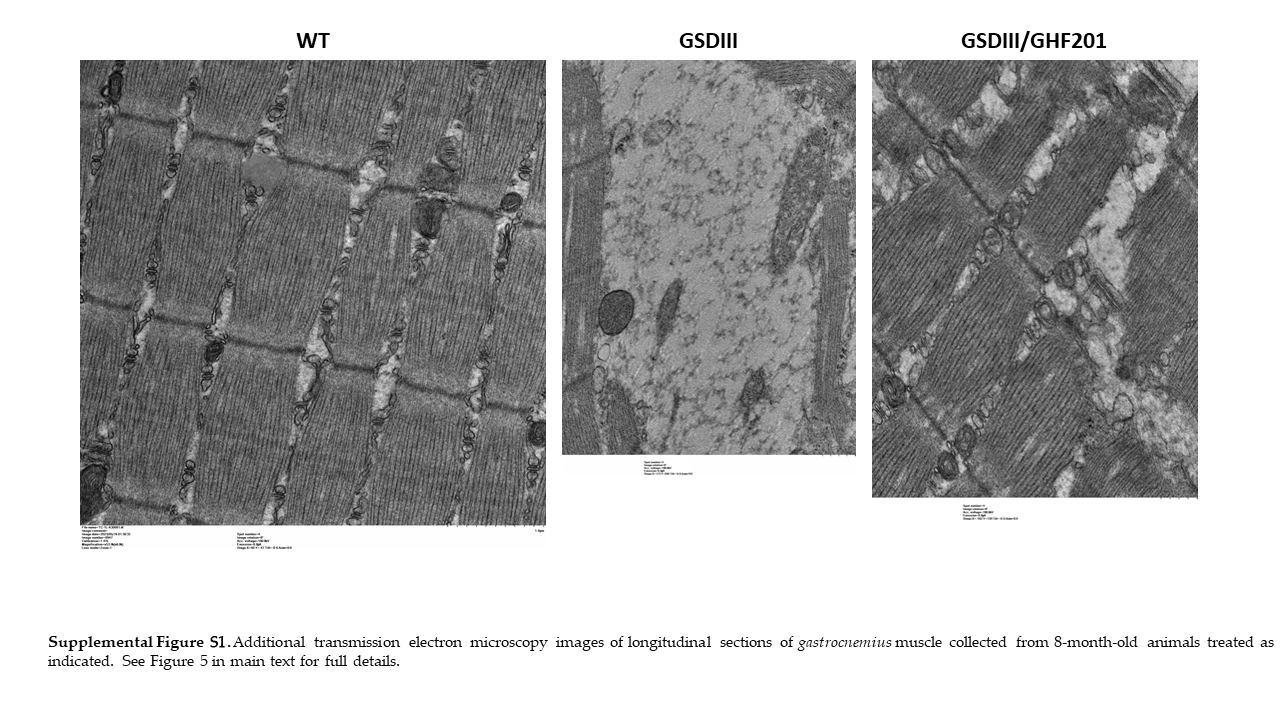

Supplement: Supplementary file 1 [file biomolecules-14-00893-s001.zip › biomolecules-3068121-supplementary.tif]
